# Inorganic Fillers and Their Effects on the Properties of Flax/PLA Composites after UV Degradation

**DOI:** 10.3390/polym15153221

**Published:** 2023-07-28

**Authors:** Moumita Sit, Saeid Dashatan, Zhongyi Zhang, Hom Nath Dhakal, Moussa Khalfallah, Nicolas Gamer, Jarren Ling

**Affiliations:** 1 Advanced Polymers and Composites (APC) Research Group, School of Mechanical and Design Engineering, University of Portsmouth, Portsmouth PO1 3DJ, UK; moumita.sit11@gmail.com (M.S.); saeid.dashatan@gmail.com (S.D.); zhongyi.zhang@port.ac.uk (Z.Z.); jarrenling77@gmail.com (J.L.); 2 Kairos, 1 rue des Senneurs, ZI du Moros, 29900 Concarneau, France; moussa@kairos-jourdain.com (M.K.); developpement@kairos-jourdain.com (N.G.)

**Keywords:** biocomposite, flax fibre, UV degradation, filler, mechanical properties

## Abstract

The present investigation seeks to assess the impact of fillers on the mechanical characteristics of entirely biodegradable composites, introducing an advanced solution to fulfil long-term durability demands within point-of-purchase (POP) industries. The inclusion of calcium carbonate (CaCO_3_) fillers on the various properties of the flax fibre-reinforced composites, after accelerated irradiation in an ultraviolet (UV) radiation exposure has been investigated in the present study. Different types of flax fibre-reinforced poly lactic acid (PLA) biocomposites (with and without filler) were fabricated. The mechanical (tensile and flexural), and physical properties of the specimens were assessed after 500 h of exposure to accelerated UV irradiation of 0.48 W/m^2^ at 50 °C and were compared with those of the unexposed specimens. The results indicate that the presence of the inorganic filler significantly improved the performance of the biocomposites compared to the unfilled biocomposites after UV exposure. After adding 20% of fillers, the tensile strength was increased by 2% after UV degradation, whereas the biocomposite without filler lost 18% of its strength after UV exposure. This can be attributed to the change in the photo-degradation of the PLA due to the presence of the CaCO_3_ filler, which acts as a safeguard against UV light penetration by creating a protective barrier. The scanning electron microscopy (SEM) images of the degraded specimen surface show substantial difference in the surface topography of the composites with and without fillers.

## 1. Introduction

In recent years, there has been a remarkable surge in the utilisation of natural fibres, biopolymers, biofilms, and bio-based composites across a wide range of versatile applications, including aerospace, automotive, and household products, signifying a notable shift towards more sustainable and eco-friendly materials [1]. It has been recognised that the environmental degradation (physical, chemical, and biological process or a combination of all) of natural fibres and polymers is one of the main concerns of the biocomposite materials that lead to the premature failure of the material. There are several factors that commonly cause degradation, including thermal degradation caused by temperature; oxidative degradation caused by air; hydrolytic degradation caused by moisture; biodegradation caused by microorganisms; photo-degradation caused by light, UV, and γ irradiation; corrosion caused by chemical agents; and mechanical stress under various service conditions [2]. The damage caused by the exposure to UV radiation is one of the main drawbacks for biocomposites used in outdoor applications. UV light is a constituent of the electromagnetic spectrum present in all types of radiation. However, UV radiation can be classified into three distinct wavelength ranges: UVA, UVB, and UVC. UVA, UVB, and UVC have distinct wavelengths of 320–400 nm, 280–320 nm, and 100–280 nm, respectively [3]. Materials can be affected by both UVA and UVB radiation. However, UVB radiation is primarily utilised in laboratory experiments to simulate outdoor conditions that would cause samples to degrade over time, similarly to the effects of normal midday sun exposure. When conducting such experiments, various factors need to be taken into consideration, including the intensity of ultraviolet radiation in the environment, which can vary depending on factors such as cloud, altitude, position, and reflection of the sun [4]. The bond detachment energy of many polymers typically occurs within the UV range of wavelengths between 290 and 400 nm [5]. Chemical processes can break or join molecular chains, while UV radiation can break chains. If weak ends are present, free radicals like oxygen or water can make polymers brittle, reducing their strength. Direct sunlight can damage covalent bonds in natural fibre composites, causing several issues, such as discolouration, reduced gloss, roughness, chalking, microcracking, fragility, and weakened mechanical properties [6,7,8,9,10,11,12,13]. It can also be seen that UV ageing coupled with other ageing sources causes the degradation of the surface, which in turn enhances the diffusion of other aggressive agents (such as water) [14].

To enhance the UV resistance of natural fibre-reinforced composite materials, they can be supplemented with fillers or UV additives that have absorbent properties [15]. Adding particulate, inorganic fillers to commercial thermoplastic and thermosetting resins is a common and cost-effective method to modify properties such as stiffness, heat distortion, and mouldability [16]. Incorporating inorganic particles into polymeric materials has been shown to improve their mechanical, thermal, rheological, optical, and photo degradation properties [16,17,18,19,20]. The effectiveness of this approach depends on factors such as the type, size, content, and surface treatment of the fillers [18,19].

Several studies [12,15,21,22,23,24,25,26,27,28,29,30,31,32,33] have explored the impact of fillers on the degradation of polymer composites, e.g., lignin [23], sisal [25], ground chestnut shells [26], pistachio shell [15], and jute [27]. Several researchers have discussed the effect of both organic and inorganic fillers on the photo oxidation of different polymers such as HDPE (high-density polyethylene) [16,29,30], polypropylene [15,31,32], polystyrene [34], and polyvinyl acetate [19]. Calcium carbonate (CaCO_3_) is widely used as an inorganic filler in polymer composites due to its low cost and easy availability [12,13,24,29,35,36]. At the nanoscale, it can impart additional functionalities to the polymer matrix, including barrier properties and antimicrobial behaviour [37,38]. CaCO_3_ acts as a UV absorber, which operates by absorbing the incident UV radiation, preventing it from reaching the polymer and converting the energy, thus transforming it into a less damaging form, such as heat.

A comprehensive review of the published literature reveals that several studies have been carried out regarding the effect of fillers in terms of the photo-degradation, thermal properties, and surface modification of the polymers. Only a few researchers [15,35] reported the effect of fillers on the mechanical properties of the natural fibre-reinforced composites. However, to date, the effect of CaCO_3_ fillers on the mechanical properties of the flax fibre-reinforced PLA composites subjected to UV degradation has not been reported in the published literature, to the best of authors’ knowledge.

The present study aims to evaluate how fillers affect the mechanical performance of fully biodegradable composites, which can meet long-term durability requirements in point-of-purchase (POP) sectors. Sustainable and fully green biocomposites made from non-woven flax fibres and polylactic acid (PLA) with and without fillers were developed to address this research gap. The study examines the effects of accelerated UV irradiation on the properties including mechanical (tensile and flexural) and physical (surface hardness, discolouration, the loss of gloss, and surface roughness) of the biocomposites developed for this study. To imitate actual service conditions, the biocomposite samples underwent 0.48 W/m^2^ UVB irradiation at 50 °C for a duration of 500 h. The mechanical and physical properties of the biocomposites were also compared to those of conventional materials like FOREX™, an expanded polyvinylchloride (PVC) panel for indoor and outdoor use. Scanning electron microscopy (SEM) was used to analyse the degraded surfaces after UV exposure to assess the surface condition. ZEISS EVO MA10 scanning electron microscope was used to acquire the image of the fractured surface of the laminates.

## 2. Experimental

### 2.1. Materials

Four different types of biocomposites were studied in the present work and are shown in Table 1. The biocomposite materials were produced and provided by Kairos, France, and are fully compostable. Flax fibres were used as reinforcement, and PLA, as well as PLA/PBAT, was used as the matrix. The CNW is based on PLA/flax laminate, at a ratio of 30% flax and 70% PLA. The PLA used in the CNW is a pure matrix and does not contain any fillers.

The KW laminate is made with PLA and flax. PLA contains 5% TiO_2_ pigment without any other fillers.

KWF_1 and KWF_2 are composite laminates based on PLA/PBAT and flax fibres. The PLA/PBAT matrix is based on a mixture of 30% talcum and 70% CaCO_3_. The specific rate (%) of each component is described in the table below.

**Table 1:** Composition of different samples.

Sample Types	Thickness (mm)	PLA (wt.%)	PBAT %	Flax (%)	TiO_2_ (%)	Filler (%) (Talcum + CaCO_3_)	Total (%)
CNW	2.7	70.48%	0.00%	29.52%	0.00%	0.00%	100.00%
KW	2.0	74.70%	0.00%	20.31%	4.98%	0.00%	100.00%
KWF_1	2.8	46.41%	9.61%	21.55%	3.00%	19.00%	100.00%
KWF_2	2.1	37.30%	8.09%	27.66%	3.00%	24.00%	100.00%

The KW laminate has a thickness of 2.0 mm, and the layup is as follows:

[PLA film of 500 g/m^2^ + 1 layer of nonwoven neat PLA/Flax fibres of 1300 g/m^2^ + PLA film of 800 g/m^2^]. The TiO_2_ pigment was integrated into the PLA film layers.

The KWF1 laminate has a thickness of 2.8 mm, and the layup is as follows:

[PLA film of 800 g/m^2^ + 1 layer of nonwoven neat PLA/Flax fibres of 1300 g/m^2^ + PLA film of 800 g/m^2^]. The TiO_2_ pigment and the fillers were integrated into the PLA film layers, respectively in the rate of 5% and 30%.

The KWF2 laminate has a thickness of 2.1 mm, and the layup is as follows:

[PLA film of 500 g/m^2^ + 1 layer of nonwoven neat PLA/Flax fibres of 1300 g/m^2^ + PLA film of 500 g/m^2^]. The TiO_2_ pigment and the fillers were integrated into the PLA film layers, respectively in the rate of 4.8% and 45%.

The CNW laminate has thickness of 2.7 mm, and the layup is as follows:

[PLA film of 435 g/m^2^ + 7 layers of nonwoven flax of 350 g/m^2^ + PLA film of 435 g/m^2^].

The PVC sheet is 4.8 mm thick.

### 2.2. Fabrication of Laminates

**Figure 1:**
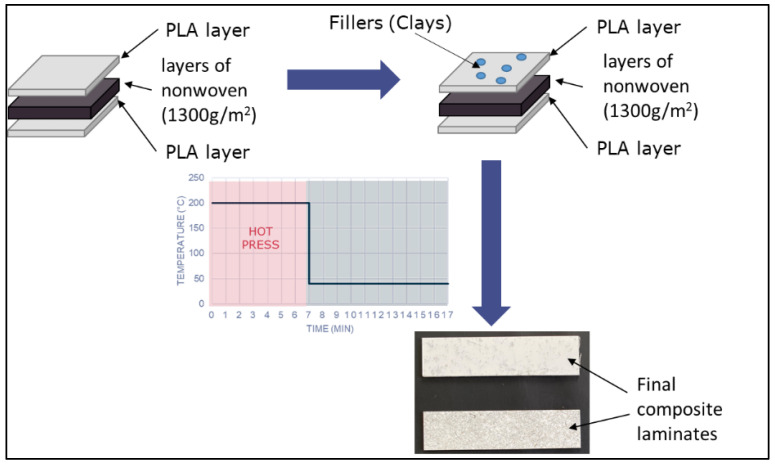
Schematic of laminate fabrication.

The laminates were fabricated by Kairos in France using a thermocompression moulding process, as shown in Figure 1.

The manufacturing of the laminates was carried out in two steps:

The first step consisted of stacking of the material layers according to the above stacking sequences and compressing these in a hot mould at 200 °C, pressed at 1 bar for 6 min.

The second step consisted of the cooling of the mould under 7 bars of pressure at 15 °C for 10 min. At this stage, the pressure was increased to enhance the impregnation of the flax fibres.

### 2.3. Specimen Preparation

The FB700 laser cutter by CadCam Technology was used to cut all manufactured composite samples, whereas a band saw cutter was used to cut the PVC samples following the British Standard EN ISO 527-2:1996 [39] for tensile tests and British Standard EN ISO 178:2010 [40] for flexural tests. To achieve a good edge finish, all samples were polished with sandpaper.

### 2.4. Accelerated UV Exposure

The samples were subjected to UV exposure using a QUV/spray accelerated weathering chamber (Q-Lab Co., Westlake, OH, USA), equipped with UVB-313 fluorescent lamps with a maximum peak at 313 nm. The UVB radiation level was set at 0.48 W/m^2^ at 310 nm wavelength and a temperature of 50 °C. The accelerated aging process with UV light was carried out for 500 h, with visual observations and surface roughness measurements taken at intervals of 100 h.

### 2.5. Physical Characterisation

#### 2.5.1. Colour Measurement

A spectrophotometer (Sheen Spectromatch Gloss Colorimeter) was used to measure the discoloration of the samples after 500 h of UV exposure. The colour of the initial specimens was compared with that of the exposed specimens. The spectrophotometer measured four characteristics: L*, a*, b*, and gloss. The L* parameter was used to describe the surface in a range of black and white, where a value of zero indicated black and a value of one hundred indicated pure white. The a* parameter indicated whether the colour was red or green, with positive values indicating red and negative values indicating green. The b* parameter indicated whether the colour was yellow or blue, with positive values indicating yellow and negative values indicating blue. The final parameter measured the gloss of the surface, with a higher value indicating a shinier surface and a lower value indicating a duller surface. The colour differences (ΔE*) of the specimens were calculated using Equation (1), as specified in the ASTM D2244 standard [41]:

(1) $$∆E^{*}=\sqrt{\left[{\left({∆L}^{*}\right)}^{2}+{\left({∆a}^{*}\right)}^{2}+{\left({∆b}^{*}\right)}^{2}\right]}$$

where ΔL*, Δa*, and Δb*, respectively, represent the changes in the initial values of L*, a*, and b* at the end of the aging period.

#### 2.5.2. Surface Morphology

SEM images were obtained from the composite samples before and after UV exposure using a ZEISS EVO MA10 scanning electron microscope (Oberkochen, Germany). To make the specimens electrically conductive, a Quorum Q150R S coater (San Jose, CA, USA) was used to prepare their surfaces with gold coating.

### 2.6. Mechanical Testing

#### 2.6.1. Tensile and Flexural Testing

Tensile and flexural tests were conducted using a Zwick/Roell Z030 (Ulm, Germany) universal machine in pull-to-break and bend-to-rupture modes, respectively, with a crosshead speed of 2 mm/min. The testing was carried out in accordance with standard procedures for tensile [39] and flexural [40] characteristics. To examine the impact of environmental ageing, at least five samples of each material with and without UV exposure were tested and averaged. All tests were performed at room temperature.

#### 2.6.2. Surface Roughness Measurement

Surface roughness was evaluated using the Mitutoyo Surface Roughness Tester (Aurora, IL, USA) to assess any changes in texture resulting from UV exposure. The R_a_ parameter, representing the average surface roughness, was used to quantify the impact on the samples’ properties.

#### 2.6.3. Shore Hardness Measurement

The shore hardness of a material was measured using the Shore D durometer, which applies a defined spring force indenter to the surface. The measurement was taken at various parts of the samples, and an average was computed.

## 3. Results and Discussions

### 3.1. Effect of UV Exposure on the Surface Colour

**Figure 2:**
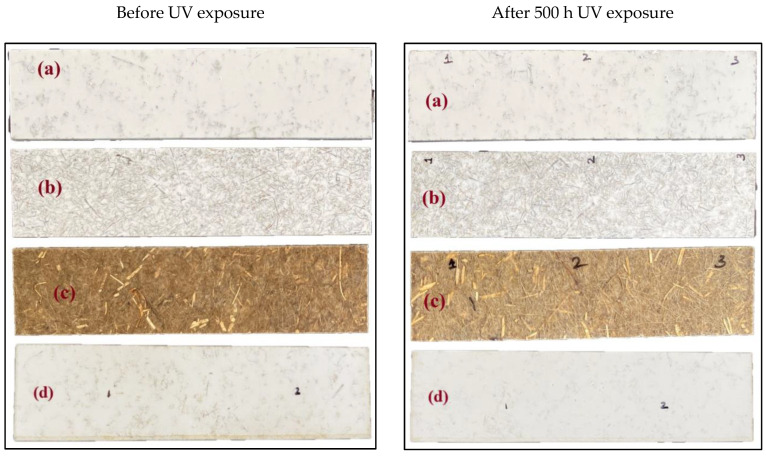
Appearance of samples before and after UV exposure (**a**) KWF_1; (**b**) KW; (**c**) CNW; (**d**) KWF_2.

Figure 2 depicts the visual appearance of the samples before and after UV exposure. The CNW sample exhibits significant changes in the appearance in terms of colour change and loss of gloss.

**Figure 3:**
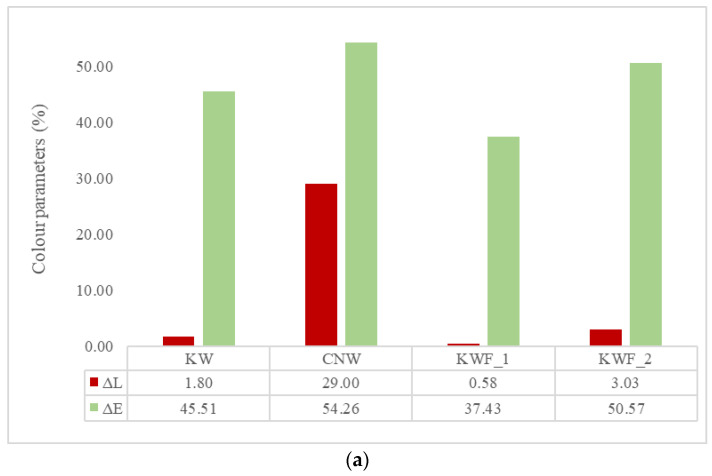

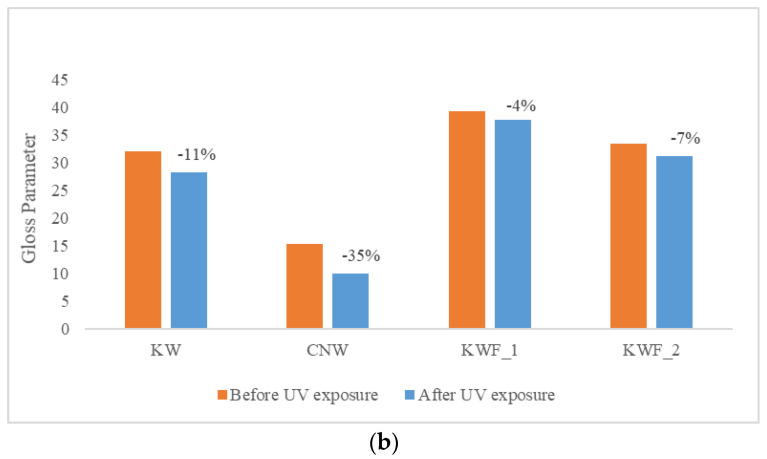
(**a**) Lightness (ΔL*) and overall colour change (ΔE*) of different samples after UV exposure; (**b**) the change in gloss parameter of different samples before and after UV exposure.

The changes in colour and gloss parameter of all the samples are shown in Figure 3a,b, respectively. It can be noted that the CNW exhibits highest amount of lightening and change in colour after UV exposure. However, the change in lightness (ΔL*) is insignificant for all other samples. The lightening of the CNW samples can be attributed to the scattered reflectance due to increased surface roughness and photo-oxidation that occurred because of polymer degradation. The overall colour change (ΔE*) is substantial for all the samples. The presence of additives and fillers did not exhibit any significant influence on the overall colour change of the composite samples during UV irradiation. The TiO_2_ particles present in the polymer act as inner screens for photo products. Since TiO_2_ acts as highly absorbing additive, the photooxidative phenomena is only limited to the surface of the samples.

### 3.2. Effect of UV Exposure on the Surface Morphology

**Figure 4:**
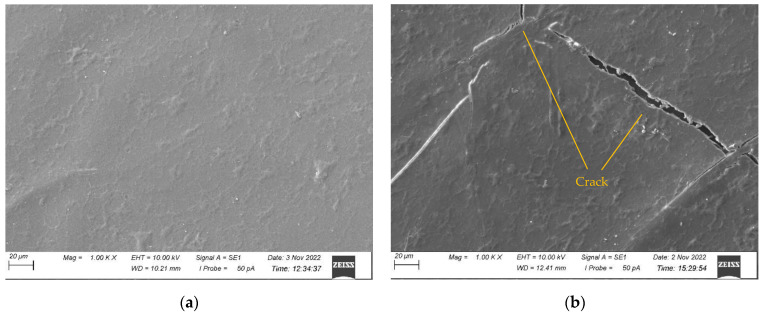
SEM micrographs of CNW samples (**a**) before UV exposure and (**b**) after 500 h of UV exposure.

**Figure 5:**
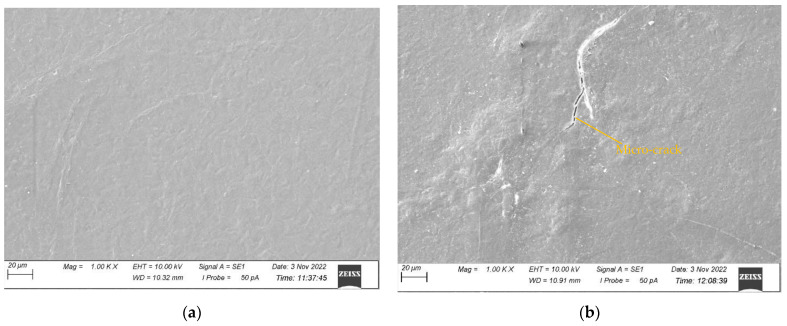
SEM micrographs of KW samples (**a**) before UV exposure and (**b**) after 500 h of UV exposure.

**Figure 6:**
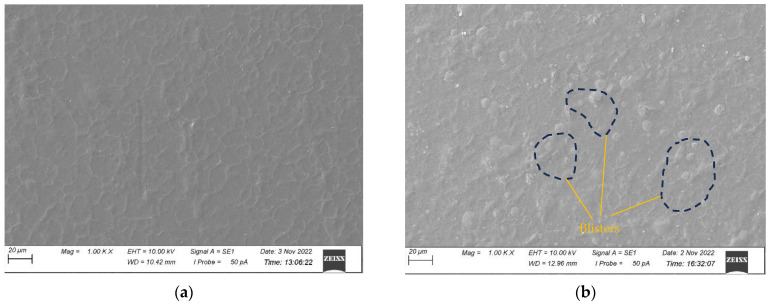
SEM micrographs of KWF_1 samples (**a**) before UV exposure and (**b**) after 500 h of UV exposure.

**Figure 7:**
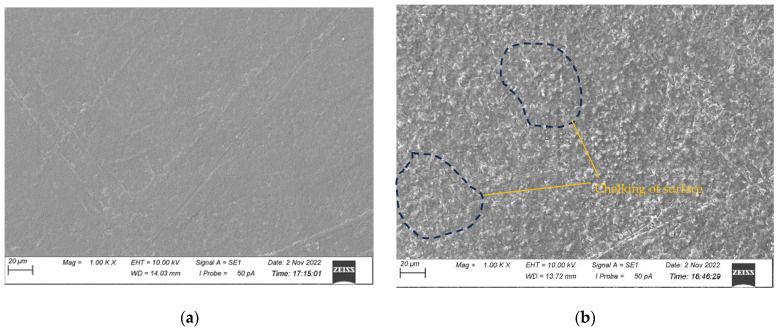
SEM micrographs of KWF_2 samples (**a**) before UV exposure and (**b**) after 500 h of UV exposure.

The surface morphologies of all the samples before and after 500 h of UV exposure were characterised by SEM and presented in Figure 4, Figure 5, Figure 6 and Figure 7 for CNW, KW, KWF_1, and KWF_2, respectively. Before UV exposure, the surfaces of the KW, KWF_1, and KWF_2 appeared to be smooth. It was observed that after 500 h of UV exposure, large cracks developed on the surface of the CNW. Exposure to UV irradiation increases crystallinity and reduces molecular weight. Therefore, surfaces tend to shrink, which ultimately leads to the development of surface cracks. For KW, due to the addition of a white additive (TiO_2_), fewer and smaller cracks were observed, along with blisters, after UV exposure. This phenomenon can be attributed to the photo-stabilisation property of TiO_2_. The filler particles absorb the UV radiation and thus slow down the photo-degradation of the polymer. For KWF_1 (19.23% of fillers), the surface of the samples after UV exposure remained relatively smooth, despite having developed microblisters. However, for KWF_2 (45% fillers), the SEM micrograph exhibited the severe chalking of the surface along with several microcracks. The formation of the microcracks could be caused due to the chemi-crystallisation, in which polymer chain scission occurs in amorphous regions [42]. It can be seen from the SEM micrographs of KWF_2 before and after UV exposure that erosion occurred around the CaCO_3_ particles.

### 3.3. Effect of UV Exposure on Tensile and Flexural Properties

The tensile and flexural properties of the composite samples are characterised both before and after 500 h of UV exposure. The tensile modulus and strength of the test samples before UV exposure are summarised in Table 2. The average flexural modulus and strength of the test samples before UV exposure are presented in Table 3. It can be seen from both Table 2 and Table 3 that the KWF_2 has the lowest tensile as well as flexural properties. It is also evident from Table 1 and Table 2 that the presence of additive (TiO_2_) and fillers (CaCO_3_) has a significant effect on the modulus of the composites. The presence of TiO_2_ particles caused an increase in both the tensile and flexural modulus in the KW composite. The tensile modulus of the KW was increased by 37% and the flexural modulus was increased by 5% compared to the CNW composite sample. This is because of the presence of rigid powder particles in the PLA, which provided resistance against the movement of polymer elastic segments (the filler was concentrated in an amorphous polymer phase) during the stretching process, thus increasing the modulus. This result is in agreement with the previously published research on poly(ethylene-co-1-octadecene) composites with TiO_2_-based nanoparticles [43]. However, the effect of the TiO_2_ particle on the tensile and flexural strength is negligible, as the neutral filler provided no particular reinforcing action.

The quantity of the filler in the polymer matrix also affected the mechanical properties of the composites. When compared with CNW, the tensile modulus was increased by 12% for KWF_1 with 20% of fillers, whereas for KWF_2, with 45% of fillers, the tensile modulus was decreased by 22%. The tensile strength was decreased for both KWF_1 and KWF_2 by 19% and 56%, respectively. Similar trends have been observed for flexural properties. Therefore, for achieving the optimal performance of composite materials, the optimal amount of fillers should be added.

**Figure 8:**
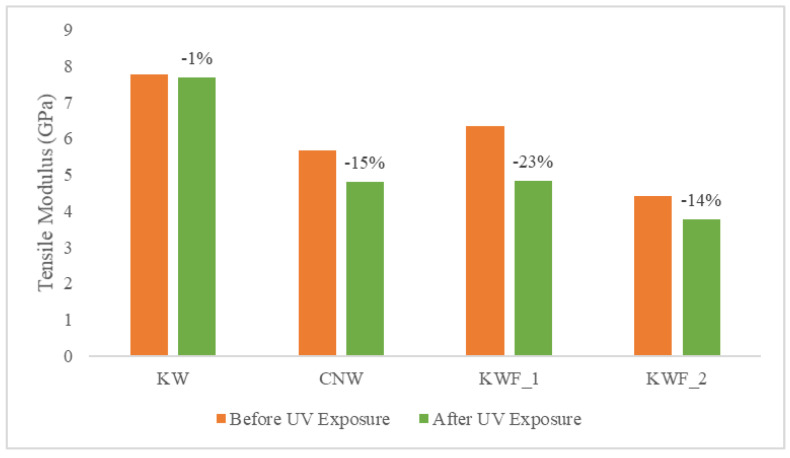
Tensile modulus for different materials before and after UV exposure.

**Figure 9:**
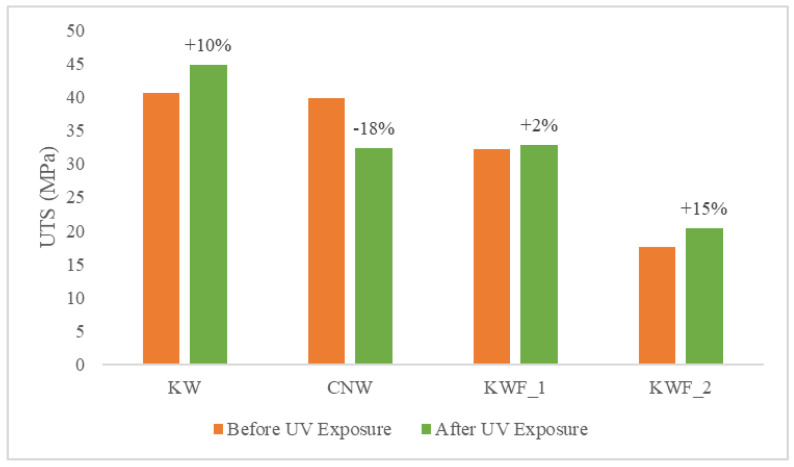
Tensile strength for different materials before and after UV exposure.

**Figure 10:**
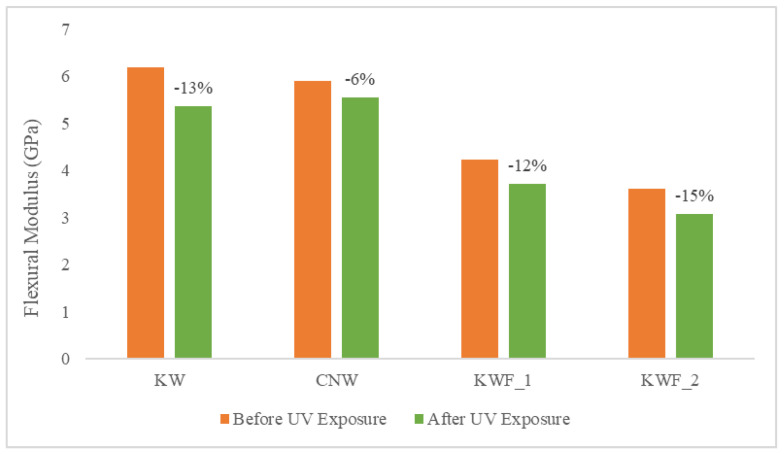
Flexural modulus for different materials before and after UV exposure.

**Figure 11:**
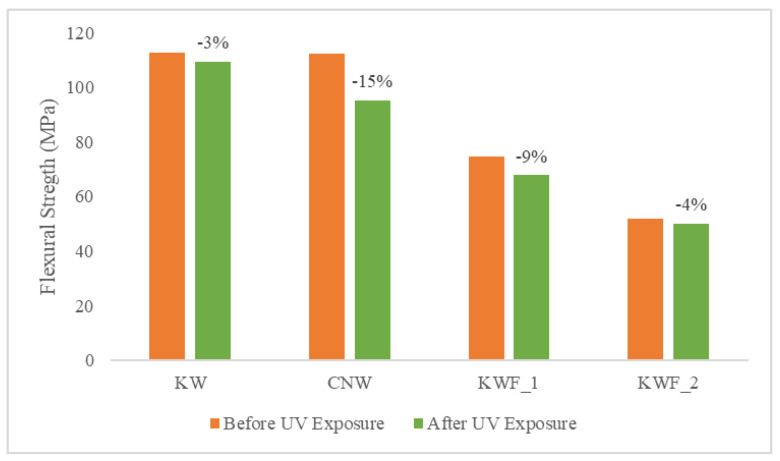
Flexural strength for different materials before and after UV exposure.

The comparison of tensile modulus and the ultimate tensile strength of the test samples before and after UV exposure are shown in Figure 8 and Figure 9, respectively. The change in flexural modulus and flexural strength of the test samples for different exposure conditions are depicted in Figure 10 and Figure 11. After UV exposure, considerable degradation was observed regarding the mechanical strength of the CNW. This can be attributed to the photo degradation of the PLA matrix. Exposure to UV irradiation increases crystallinity and reduces molecular weight [25]. Therefore, the surface tends to shrink, which ultimately leads to the development of surface cracks. These surface cracks act as micronotches, leading to the failure of material under the mechanical load, thus causing the deterioration of mechanical properties. The tensile modulus of KWF_1 and KWF_2 has also reduced significantly after UV exposure, whereas the change is negligible for KW samples. However, KW, KWF_1, and KWF_2 exhibit an increase in tensile strength. Compared to CNW, the loss of flexural strength is also insignificant for KW, KWF_1, and KWF_2. This improvement in mechanical strength can be mainly ascribed to the photo-stabilisation property of TiO_2_ and CaCO_3_. The additive and filler particles absorb UV radiation, thus retarding the photochemical process that occurs during the irradiation [34].

Although the present study was conducted under 500 h of UV exposure, the samples displayed resilience and the degradation under UV was minimal. However, if the exposure duration were increased to 1000 h, for example, it can be envisaged that the degradation could be more severe. In addition, the effect of the fillers would be more noticeable under longer durations of UV exposure.

### 3.4. Effect of UV Exposure on Surface Roughness

**Figure 12:**
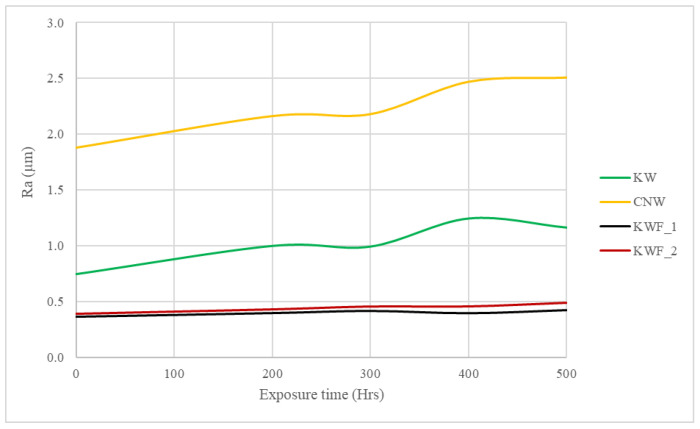
Surface roughness (Ra) measurement of the samples at different time intervals after UV exposure.

Figure 12 shows the changes in surface roughness with increasing exposure time. It can be seen that there is insignificant change in the roughness of KWF_1 and KWF_2. However, the CNW and the KW samples exhibited increased surface roughness when compared to the initial stages. The increase in surface roughness was caused by UVB radiation, which changes the matrix and leads to the formation of new cracks or the expansion of pre-existing surface defects.

**Figure 13:**
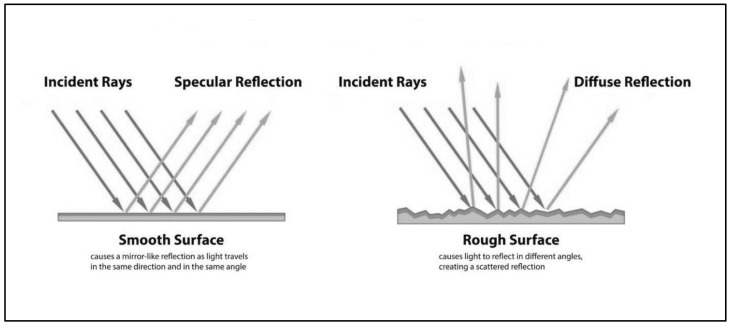
Relationship between surface roughness and gloss.

It was also observed that as the surface roughness increases after UV exposure, the gloss decreases. For example, the CNW exhibits the maximum increase in the surface roughness as well as the maximum reduction in gloss parameter after UV exposure. A similar trend was observed for other samples as well. This correlation of gloss and surface roughness is attributed to the reflection of light. As depicted in Figure 13, incident light is completely reflected at a certain angle by an extremely smooth surface, demonstrating a high gloss effect, whereas for a rough surface, light is reflected in a scattered form instead of total reflection, consequently causing a matting effect [44].

### 3.5. Effect of UV Exposure on Shore Hardness

Hardness refers to a material’s surface resistance against indentation and is influenced by the flexibility and mobility of the polymer chain structure. The values in Table 4 display the Shore D durometer measurements for the hardness of various samples. It is evident that UV exposure has a minimal effect on the Shore hardness. The hardness of the CNW was slightly increased after exposure to UV, which may be due to increased crystallinity. The change in hardness for other composite samples is negligible.

## 4. Conclusions

The influence of inorganic fillers (TiO_2_ and CaCO_3_) on the physical and mechanical properties of flax fibre-reinforced PLA biocomposites before and after UV exposure were investigated in the present study. The samples were exposed to UVB irradiation of 0.48 W/m^2^ for 500 h. The presence of the fillers was found to increase the mechanical and physical properties significantly. The addition of filler particles also enabled us to achieve smoother and glossier surface finishing. Tensile and flexural moduli were improved after the addition of fillers. It was also observed that the composites with filler particles exhibited improved tensile strength after UV exposure. The change in flexural strength after UV aging was also insignificant for the samples with fillers when compared to the samples without fillers. A 4.98 wt% TiO_2_-filled KW composite displayed the highest tensile and flexural properties. The observations from the results indicate that TiO_2_ and CaCO_3_ are effective UV stabilisers that can significantly prolong the service life of the biocomposites in terms of mechanical (tensile, flexural, and hardness) and physical (surface roughness, colour stabilisation) performance. Addition of 24 wt% CaCO_3_ increases the tensile strength by 15% after UV exposure. However, it has also been noted that the quantity of fillers also influences the modulus and strength of the resulting composites. Therefore, it may be concluded that with optimal amount of fillers, a sustainable biocomposite with optimal performance can be achieved for outdoor application.

## Figures and Tables

**Table 2:** Tensile test results of different materials.

Material	Mean Tensile Modulus (GPa) ± SD ^#^	Mean UTS (MPa) ± SD ^#^
CNW	5.67 ± 0.51	39.81 ± 5.29
KW	7.78 ± 0.49	40.52 ± 4.90
KWF_1	6.34 ± 0.29	32.22 ± 2.76
KWF_2	4.40 ± 0.37	17.63 ± 1.61

^#^ SD represents the standard deviation of the mean.

**Table 3:** Flexural test results of different materials.

Material	Flexural Modulus (GPa) ± SD ^#^	Flexural Strength (MPa) ± SD ^#^
CNW	5.90 ± 1.46	112.35 ± 5.13
KW	6.19 ± 0.16	112.81 ± 4.40
KWF_1	4.22 ± 0.23	74.78 ± 1.54
KWF_2	3.62 ± 0.25	51.99 ± 3.34

^#^ SD represents the standard deviation of the mean.

**Table 4:** Values of Shore D hardness before and after UV exposure for different materials.

Material	Before UV Exposure± SD ^#^	After UV Exposure± SD ^#^
CNW	85.0 ± 0.70	87.0 ± 0.50
KW	84.8 ± 0.54	85.0 ± 0.48
KWF_1	81.6 ± 0.55	81.8 ± 0.38
KWF_2	78.5 ± 0.50	78.0 ± 1.22

^#^ SD represents the standard deviation of the mean.

## Data Availability

The data that support the findings of this study are available on request from the corresponding author.
